# Cellular Simulation for Distributed Sensing over Complex Terrains

**DOI:** 10.3390/s18072323

**Published:** 2018-07-17

**Authors:** Tuyen Phong Truong, Bernard Pottier, Hiep Xuan Huynh

**Affiliations:** 1 Faculty of Sciences/Lab-STICC, University of Brest/UMR CNRS 6285, 29238 Brest, France; 2 College of Information & Communication Technology, Can Tho University, Can Tho 900000, Vietnam; hxhiep@ctu.edu.vn

**Keywords:** cellular automata, complex terrain, LoRa, parallel processing, radio signal propagation

## Abstract

Long-range radio transmissions open new sensor application fields, in particular for environment monitoring. For example, the *LoRa* radio protocol enables connecting remote sensors at a distance as long as ten kilometers in a line-of-sight. However, the large area covered also brings several difficulties, such as the placement of sensing devices in regards to topology in geography, or the variability of communication latency. Sensing the environment also carries constraints related to the interest of sensing points in relation to a physical phenomenon. Thus, criteria for designs are evolving a lot from the existing methods, especially in complex terrains. This article describes simulation techniques based on geography analysis to compute long-range radio coverages and radio characteristics in these situations. As radio propagation is just a particular case of physical phenomena, it is shown how a unified approach also allows for characterizing the behavior of potential physical risks. The case of heavy rainfall and flooding is investigated. Geography analysis is achieved using segmentation tools to produce cellular systems which are in turn translated into code for high-performance computations. The paper provides results from practical complex terrain experiments using LoRa, which confirm the accuracy of the simulation, and scheduling characteristics for sample networks. Performance tables are produced for these simulations on current Graphics Processing Units (GPUs).

## 1. Introduction

Climate change and natural evolution seriously impact several social and economic aspects, including living conditions, human health, and development. Autonomous observation is a key point for understanding evolution and the management of territories. This is achieved using small stations equipped with processors, sensors, transceivers, and power supplies. Distributed sensors sample physical behavior. They enable analyzing environmental changes and to predict short-term and medium-term transformations.

Wireless Sensor Networks (WSNs) offered several frameworks to connect sensors. Radio standards such as 802.15.4 [1] propose short range mesh connected topologies suitable for indoor or local deployments. Recent innovations include low power long-range radio systems, such as LoRa [2] or Sigfox [3]. Long range means covering a large surface, more risk during transmissions, long signal latencies, and more difficulties coming from the ground topology. *Star organization*, where a central node addresses remote sensors is the more natural technique to collect data on surfaces within a range of ten thousand square meters.

Complex geographic areas such as shores or islands, hills, valleys oppose physical difficulties to the radio propagation. At the sensor level, the *coverage* also defines zones where information is accessible, whatever it may be, wildlife including insects, physical elements such as water or gas. Therefore, a *precise coverage computation* is critical for monitoring efficiency which matches *radio connectivity objectives*, and sensing accuracy in regards to physical phenomena. This article describes principles of a general method based on geo-localized cellular systems. It explains how a radio coverage or a physical coverage can be computed from the same framework, allowing for obtaining a better match between observation engines and observed phenomena.

**Figure 1:**
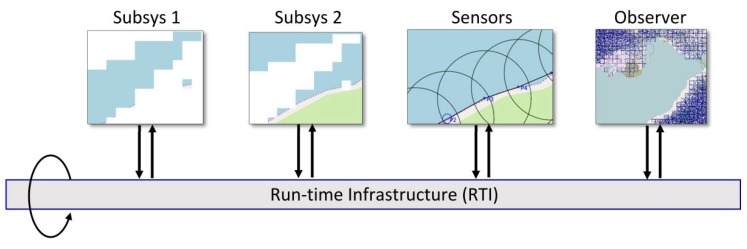
Federation of two complementary cellular subsystems, a sensor network, and some display for observation. A software bus (RTI) provides services for data exchanges, sequencing and synchronization. This framework is called *High Level Architecture*, standardized as IEEE Std 1516–2000 [5].

A cellular approach was chosen to address the variety of entities appearing in physical situations. Geography variation is one of them, with elevation accidents, rivers, lakes, forest, and shores. By fragmenting geography into cells, it becomes possible to isolate different behaviors, to simulate, then to reassemble these independent behaviors using higher level simulation paradigms, as shown in Figure 1 and [4].

The approach of fine grain cellular structure and coarse grain partitioning appears to be flexible, allowing different components to coordinate. It is also compatible with geographic information since each cell is localized.

The introduction firstly gives an overview on principles behind three components in the methodology: (i) the terrain analysis (Section 1.2), (ii) the signal propagation and coverage (Section 1.3), and (iii) the representation of physical phenomena behaviors (Section 1.4). Generic algorithms for sensing deployments can be built in relation to metrics for these components. This section will refer to a grid of points having geometric and geographic coordinates. Preliminary examples present a large region with mountains and creeks along the seashore, likely to be equipped with sensors. This level of explanation matches application needs at an engineering level.

Further sections will detail tool principles, with the presentation of the cellular methodology (Section 2), then algorithms which simulate the physical case of flooding (Section 3), and long-range radio propagation (Section 4). Appendix A will present validation results obtained by these algorithms.

### 1.1. Related Work

Use of a cellular automata approach for modeling of natural phenomena in terms of terrain complexity has attracted the interest of several scientists. Climate change impacts and sea level rise, leading to severe weather, salinity intrusion and natural disasters are described in [6,7,8,9,10]. From these studies, proper plans and forethought can support adaptation and resilience. In addition, other publications have investigated hydrological modeling with respect to homogeneous of landform types, for example, surface water flow simulation, complex river system modeling, and flash flood forecasting [11,12,13]. Solutions for problems related to land use, land erosion, and landslide have been proposed by [10,14,15]. Another related research topic is environmental pollution such as oil spill, air pollution, and so on [16,17]. Many natural disasters (e.g., volcano, earthquake, tsunami, snow avalanches, etc.) could be forecasted through computer programs based on cellular technology [18,19,20,21]. Modeling wildlife propagation and urban in relation to food, environmental variables are also research fields for CA [22,23,24].

### 1.2. A Characterization of Terrain Complexity

Sensor investigation in a zone almost certainly starts with a glance at a geographic map or an aerial photography. If the zone includes mountains, hills, rivers, or shores, two questions will arise, about the sensing point locations, the reachability of an infrastructure network, and the adequacy with the sensing objectives. Design tools will help to take into account the geographic characteristics and possible obstacles to signal propagation, while keeping track of these objectives. Sensing systems are application specific, point to point, and differ a lot from mobile radio communication based on regular coverages.

**Figure 2:**
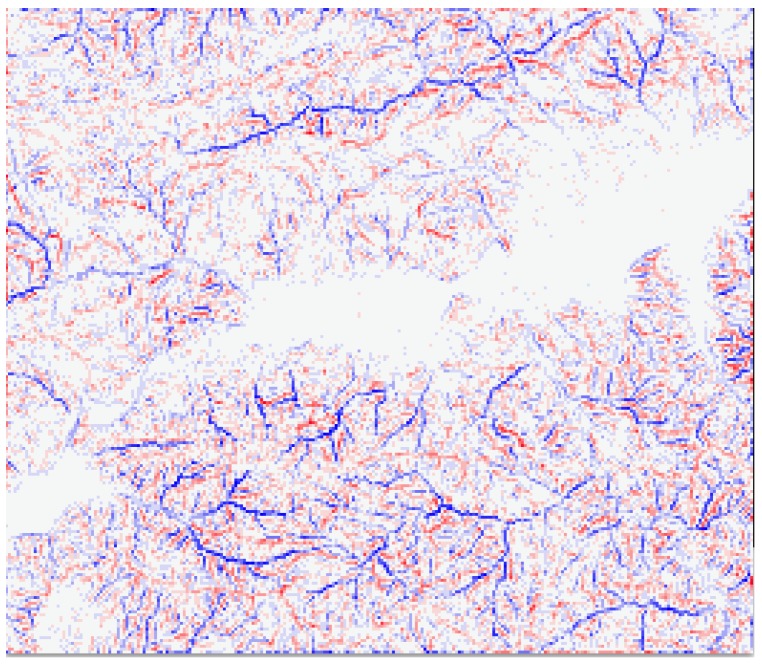
TPI terrain complexity for the river Soummam in Algeria (see also Figure 5). Red lines represent higher points, difficult to overcome and blue lines are for lower points, difficult to reach. The white zone signals a flat ground without remarkable obstacles, the case of Soummam banks. This grid is 262 × 226 points, representing 30 × 25 km.

*Terrain complexity metrics* allow for measuring ground irregularity, and offer the possibility to split and isolate zones of similar characteristics. Figure 2 displays complexity for a complex zone (Soummam Valley, leading to Bejaia city, the north of Algeria) based on Digital Elevation Models (DEMs) analysis.

**Figure 3:**
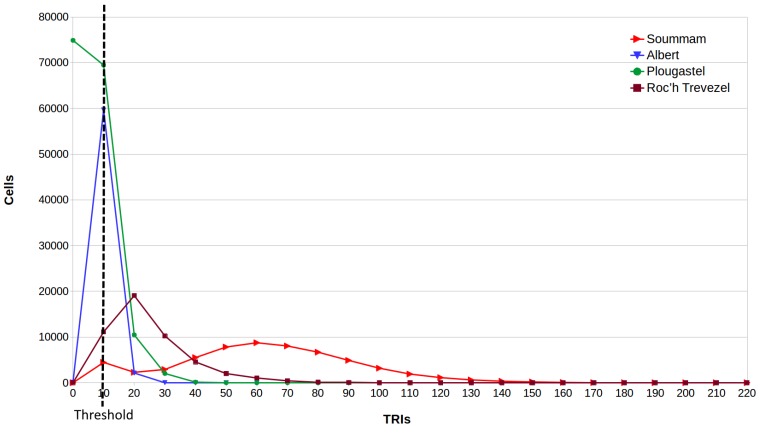
A histogram of topography ruggedness. Four zone analysis with high variability (red, the Soummam), low variability (blue, the City of Brest, with several deep valleys and the shore), a zone with an high percentage of sea surface (green, Brest bay), and medium variability with low size hills (brown, the Arrée mountains).

**Figure 4:**
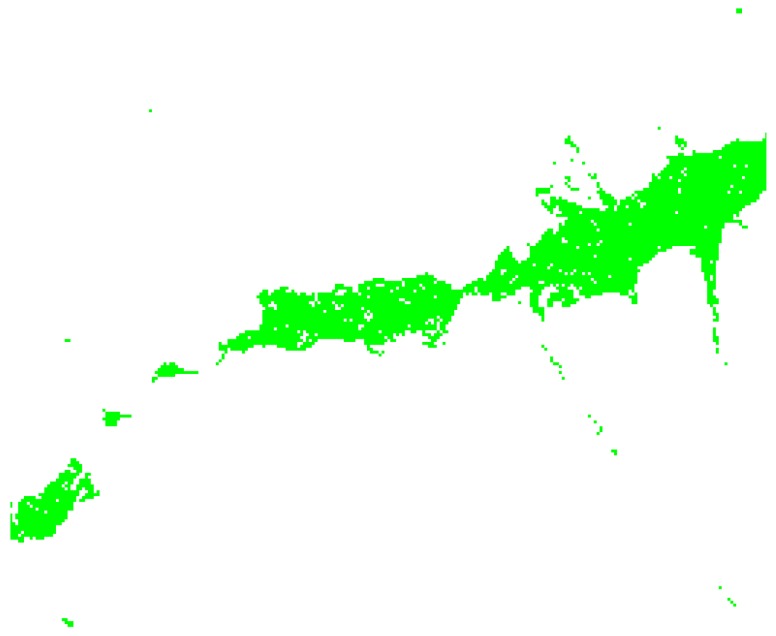
A subsystem of low terrain complexity was extracted from Figure 2. This is a flat zone around the river Soummam, with complexity below the threshold line on the left in Figure 3.

Several metrics are known for this. *Terrain Ruggedness Index* (TRI) and *Topographic Position Index* (TPI) for DEM provide a quantitative measure of surface turbulence [25]. TRI is the square root of the summed squared deviation in elevation between a cell and its eight neighbor grid cells [26]. TPI is the difference in elevation value of a center cell and the mean of eight adjacent neighbor cells [27]. Both TRIs and TPIs grids illustrate the distribution of terrain heterogeneity and may be displayed in the form of a map as shown in Figure 2. Figure 3 display analysis results for four cases, also showing how terrain characteristics allow separating concerns about sensor layouts. Partitioning the ground according to terrain complexity (as shown in Figure 4) is a preliminary operation for network layout and selection of the pair to pair radio transmissions, to be obtained through computer programs.

### 1.3. Designing for Long-Distance Radio Coverage

Setup of long-distance radio communication links is difficult if there is an obstacle in the geographical topology.

In smooth terrain areas, radio propagation can be considered as *near line-of-sight (NLoS)*. In this case, the communication distance is expressed in relation to power reduction along the path. *Free space path loss (FSPL)* equation was proposed for this aim (see Section 5 and Equation (9) in Section 5).

Concerning complex terrains, the choice of a radio position can be application context dependent, topology-dependent, or it can come from a layout algorithm. Thus, it is critical to obtain a description of the zones covered by a signal, plus other characteristics such as propagation delay from the source, and quality of the signal. Section 5 will explain how this can be done using cellular algorithms which mimic the spatial physical propagation over the terrain.

**Figure 5:**
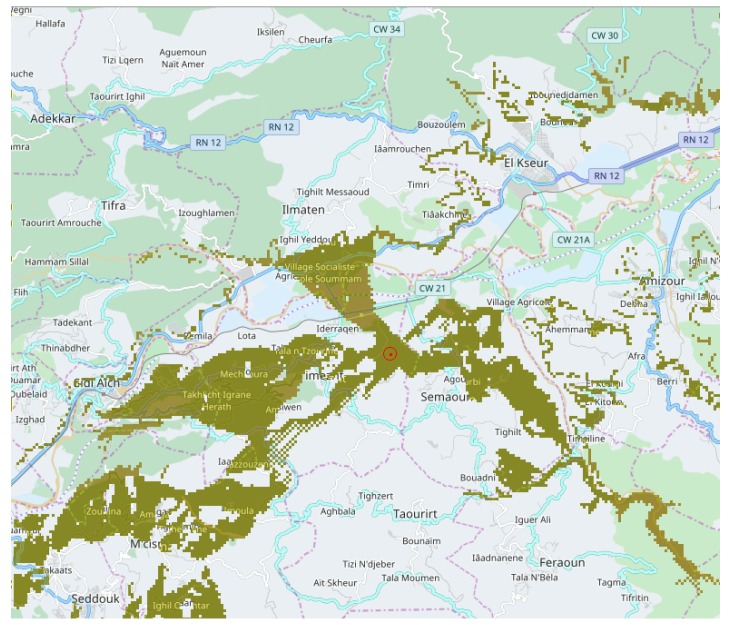
Display of a radio coverage for an emitter (red point), located above the Soummam (36.622141028, 4.799995422), elev:165.0 m. Note that geographic positions are proposed in (lat, long) form compatible with familiar map navigators. Coverage is shown in dark yellow, spreading over 30 km on the zone width. This location was chosen at random, giving a percentage of 32% grid points receiving the long-range signal.

Figure 5 illustrates the results of a coverage computation, from an expected source in the middle of the figure. The irregular yellow shape reflects the difficulty to choose source and destination given that any position between them can interrupt the signal propagation. Deploying a network of several nodes on long distances is not tractable by hand.

### 1.4. Observing Physical Phenomena

Another component for observation methodology is the necessary focus on the phenomenon to monitor. The nature of sensing varies a lot, depending on application fields. Sensing can be related to biology, climate, geology, human activities, or composition of parameters. Complex terrains will bring a complex behavior, as it is the case for radio signal propagation.

To illustrate the observation problem, let us consider heavy rain in the same region as in Section 1.3. The physical fact to be monitored is water streaming from the mountain to the river, then to the sea. Rain can be predicted from the meteorology services simulations, or just observed using radars, satellites, or ground devices. Causality for water flooding is the nature and shape of the terrain, sharing characteristics with terrain complexity and signal propagation.

**Figure 6:**
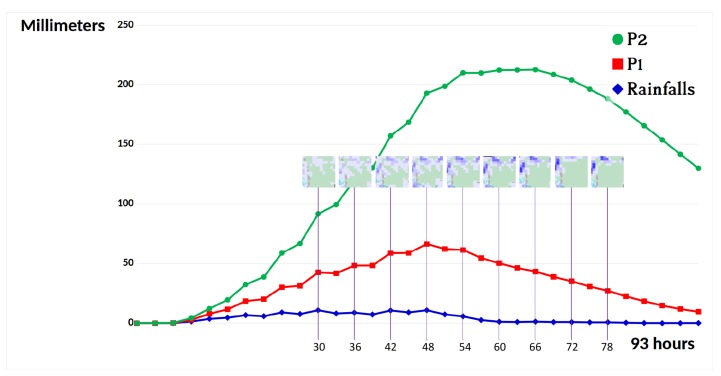
Chart of rainfalls and water levels at two positions in the Soummam river zone during a tropical storm from 13 to 16 November 2017. The blue line shows rainfalls during four days recorded every 3 h [30]. The two lines in red, green color present water level at P1, P2, respectively (also see Table 1). The distance between P1 and P2 is 300 m. Due to the significant slope of the ground surface in this complex terrain, a large amount of water was accumulated at lower points, causing a flash flood, and possibly a catastrophic landslide.

Cellular simulation presented in Section 2 will compute propagation of water given an expected, or existing rain history taken from a public service (this case), or extracted from radar networks [28] or satellite observations [29]. Figure 5 displays the water streaming from the mountain down to the river in this particular case of a heavy storm whose profile is shown in Figure 6.

**Figure 7:**
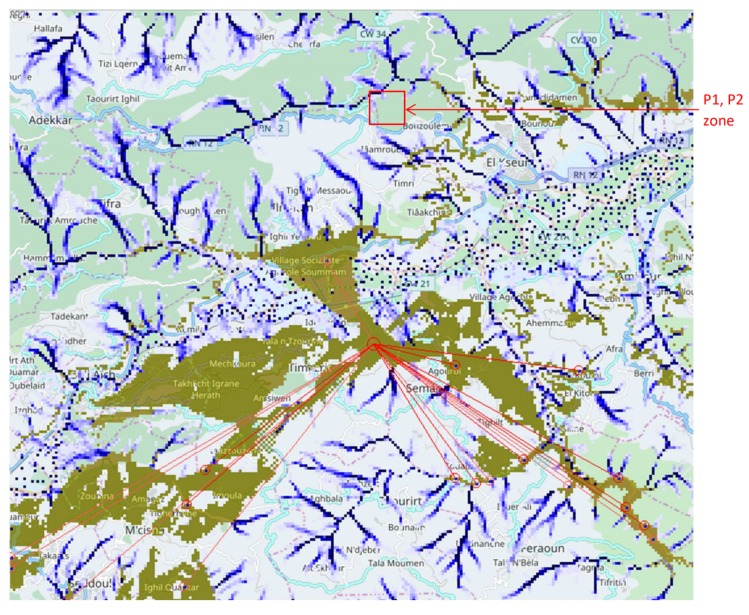
Heavy rainfalls context: a physical simulation produced positions with the risk of flash flooding (dark blue color). The communication coverage of a base station with a star network is predicted for sensor nodes monitoring level of water in these positions. An algorithm selects reachable sensor positions from a network sink, sorts, then extracts 15 positions according to the flooding result simulation. More details about accuracy are given in Section 3.

In the resulting maps, high values represent flooding and possible risks. This analysis demonstrates that simulation allows for establishing dependencies between risks and positions. Choice of real sensing positions can be produced by computer programs. For example, the control that appeared in Figure 7 can connect on radio coverage zones and obtain measures from critical positions in the rivers. The difference in communication latencies is another problem in the long range. This is also a place where computer tools can help to produce a schedule of sensor communication automatically.

The flexibility of cells embedding local parameters makes it possible to represent many physical phenomena such as ocean or river wave effects, rain effects and flooding, air or water pollution, species behavior, sound, and alerts reachable zones, etc. Parallel algorithms were designed to simulate rain flooding (Section 3) and propagation of the LoRa radio protocol (Section 5.1), the objective being to obtain high performances on these kinds of problems.

Now, the article explains the geographic space structuration based on cellular systems.

## 2. Mapping Geographic Space into Cell Systems

### 2.1. Geographic Map and User Interfaces

Several domains such as physics, medicine, or biology need sensing systems and geometric references. The case of geography includes necessary *projections* of a spherical surface into flat maps, with many options depending on applications, precision and considered location. Software tools exist for conversions [31]. Designing observation systems implies considering distances and geometry relating to applications, and therefore use of one or several reference systems to manage physical phenomena and their perceptions coherently. This section proposes an explanation of map building and displays supporting the meaning of map contents, and data abstraction for geo-localized information additionally fetched from other sources, including simulations.

**Figure 8:**
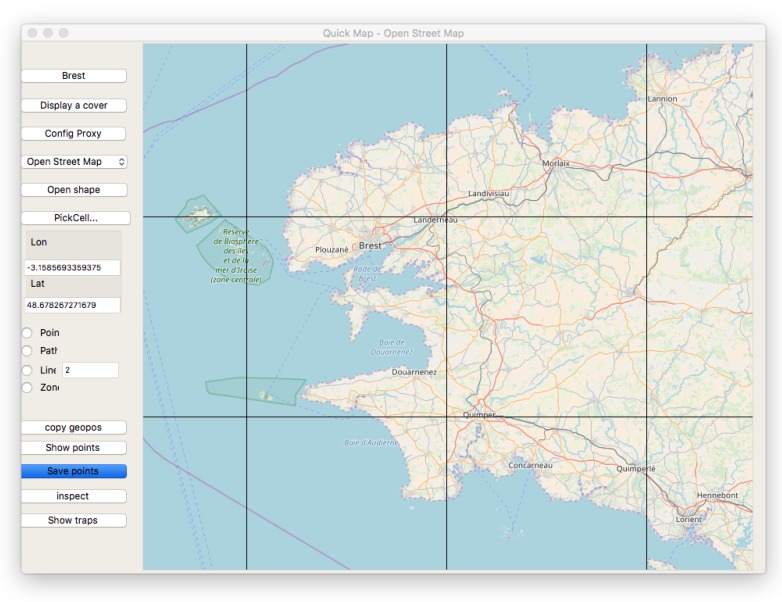
Quickmap tool [33] showing tile coverage. For this case, zoom factor is 9, and the last tile at the bottom right has *x* = 251, *y* = 177 indexes.

**Figure 9:**
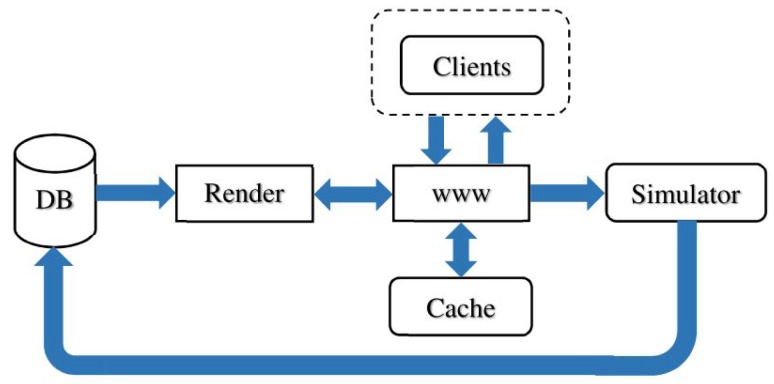
Tile map server architecture: tiles are requested from the web page server that either return a cached image or ask the rendering engine to compose it from a data base of geographical objects.

*Tiled web maps* are used to represent geographic data and to display information as flat graphics. This appears in proprietary software, or free access map systems such as OpenStreetMap. In *Tiled web maps*, the Earth is accessed by a projection rule called *Web Mercator* [32], and most often by a 3D designation mechanism *XYZ* used by web browsers and tile servers. The Z parameter provides a *zoom factor* from the whole Earth to more and more detailed view of the Earth fragments. At a given zoom factor Z, XY gives the 2D index of a tile inside the rectangle of tiles of this level Z. Based on this, tiles of graphical information are composed and delivered by servers to clients, usually in the form of 256×256 pixel images (Figure 8 presents a map composed from such tiles). These images are composed by a server rendering engine that extracts objects from a database and draws them according to some style (see Figure 9).

The browser *Quickmap* [33] supports standard map tiles, including OpenStreetMap, and a variety of other items, either for maps or aerial images. This tool also allows for describing sensor systems, communication links, and mobile trajectories, for example, Low Earth Orbit (LEO) satellites [34].

At a map browser level, users specify or observe *geometric points relative to a zoom level*. Real points are geographical positions, with coordinates specified according to the current projection system. In the case of Quickmap, they are double precision floating point numbers for latitude and longitude. Another concern is the necessity to use real distances in meters, over the Earth surface, for a task such as coverage computation. In the case of Web Mercator transformations between addressing mechanisms for *(lat, long)* absolute geographical specifications (*x*, *y*) distance in meters, (*x*, *y*) pixels, and XYZ tile access operations can be managed with formula from [35].

### 2.2. Definition of Cells

**Figure 10:**
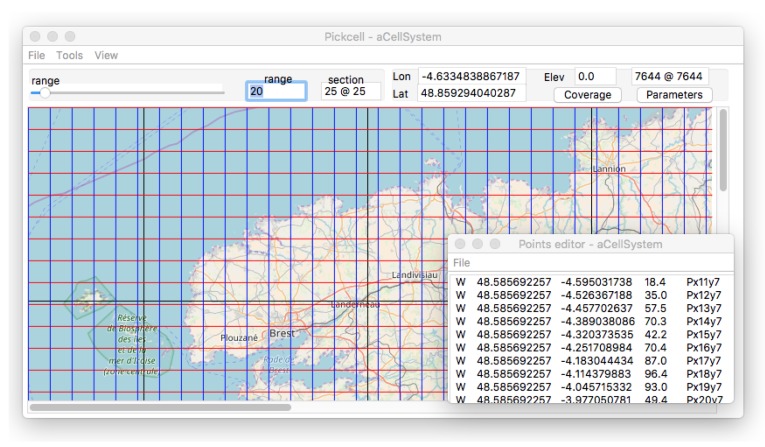
Presentation of a cell system organization over the tiles of Figure 8. Each cell has an identity produced from its location inside the window, and a geographical location. The text window bottom right also displays a plus parameter for the elevation. The cell size is 25×25 pixels, representing 7644 m.

*Geographical cells* can now be defined as *objects* grouping local data, local system behaviors, and graphic representation. This is described as an object-oriented class, as variables, and methods that are strictly local to the cells (Figure 10):

- *Cell locations* are bounded to geometric locations on maps, in relation to tile containers, and when possible to geographical locations.
- *Basic cell contents* are extracted from the map or image fragment as supported by the browser tool.
- *Cell content extensions* are obtained from external databases. Most of the cases are digital elevations, and also climate, or weather characteristics and historical data.
- *Cell size* is chosen to match a particular physical phenomenon or sensing requirements.
- *Cell behaviors* are local procedures operating on a cell state. They need to be programmed to produce simulation data that in turn can be sent back to databases, or displayed on tiles.

Examples in Section 1 illustrate how geographical abstraction, representation, and simulation can interact (Figure 2, Figure 5 and Figure 7).

Given a partitioning of a geographical zone into a cell system, we need to relate the observation system and physical behavior. The possibility to simulate physical processes will be of great help to correctly characterize a layout of sensors in connection with physical evolution.

*Cellular automata* (CA) allow for modeling physical activities as processes that exchange information and evolve according to transition rules. The cell concept binds geographical fragments to such processes whose assembly is generated automatically.

### 2.3. Cellular Automata Principles

#### 2.3.1. Synchronous Systems

Cellular automata were invented by John Von Neumann and colleagues with the aim to build a self-reproducing machine abstraction [36]. To support this goal, a two-dimensional space was configured with automata governed by a small set of states. This representation of space was used in several scientific domains and bound to physical behavior having similar properties [37]. CA can be described, and specified as a discrete space which associates cells.

**Figure 11:**
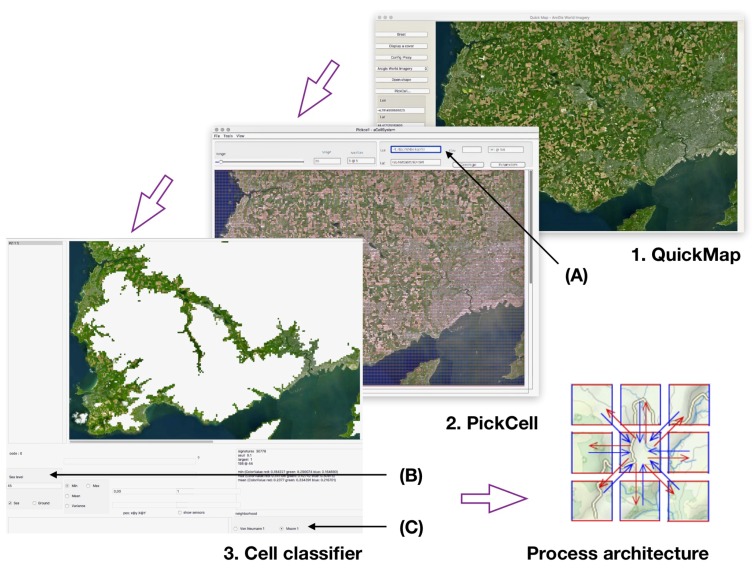
Cell synthesis flow: (1) a zone was located from a Quickmap navigation, and (2) was segmented into cells, then a subsystem was extracted filtering cells with elevations less than 45 m. (3) A cell system was generated following Moore topology. Annotations show controls for geographical positions with a cell size of 5×5 pixels representing a 191×191 m${}^{2}$ surface (**A**), classification was operated for elevation (**B**). The neighborhood was Moore, radius 1 (**C**).

In *synchronous cellular automata*, cells evolve, step by step, following a discrete time. Such CA have been described in a variety of languages and executed on specific machines [38] following four simple patterns:

- The *cellular space* is represented by an assembly of similar cells. A common notion of *neighborhood*s defines local communications following observed physical dependencies. The spatial organization can be either regular or irregular, possibly with disconnected subsystems as shown in Figure 11, item 3.
- The evolution of each cell is defined in a *set of states* as observed in a real system (quantities, colors, boolean). Change of states are operated by procedures associated with representing transition rules from step to step: $State_{t}\to State_{t+1}$.
- The *neighborhood* represents physical dependencies, for example, signal propagation, or downward flooding. These dependencies are connectivities from cell to neighbor cells. Common neighborhoods are Von Neumann and Moore with four and eight cardinal directions, respectively. Item *“Process architecture”* in Figure 11 illustrates a Moore neighborhood.
- The *transition rule* defines the behavior of each cell evolution under influence of its neighborhood and local *sensed* influences. The state of the whole cell system synchronously changes, time step by time step.

*Non deterministic* behaviors include random variation at the physical level. This was used in lattice gas simulation [39] and to represent life cycle and species evolutions [40].

#### 2.3.2. Variability in Large Systems and Asynchronism

Considering large systems, synchronism and massive data parallel execution can be an obstacle, both in behavior modeling and performances because of sparse data spaces.

Asynchronous cellular automata were proposed to represent reactive systems where events are propagated in a way similar to Communicating Sequential Processes [41]. However, in the case of physical simulation, sampling and time references are often mandatory. Variability of computation frequency can be obtained by isolation and simulation of sub-systems. This was implemented on GPUs as related in [42].

The High Level Architecture (HLA) also allows for sequencing cellular sub-systems at a different speed and to provide data exchanges between them. For example, Ref. [4] shows a countryside simulation with forest, forest fire, sensors and river pollution composed together.

Thus, we can consider the grouping of cells into synchronous subsystems as an efficient way to manage variability. Composing simulations at a high level is shown in Figure 1, and was technically discussed in [4].

### 2.4. Cellular Automata Parallel Execution Models

A sequential execution model will read states in an array $A_{t}$ for time *t*, and loop over, writing a similar array $A_{t+1}$. Once the step is completed, the two arrays are exchanged, and a new turn begins. CA is easy to parallelize preserving this behavior, grouping operations together and observing turns completion. Parallelism is mandatory because problems are large, if not huge, a lot of small cells can be critical for simulation precision, and some applications require examining very large geographical regions.

**Figure 12:**
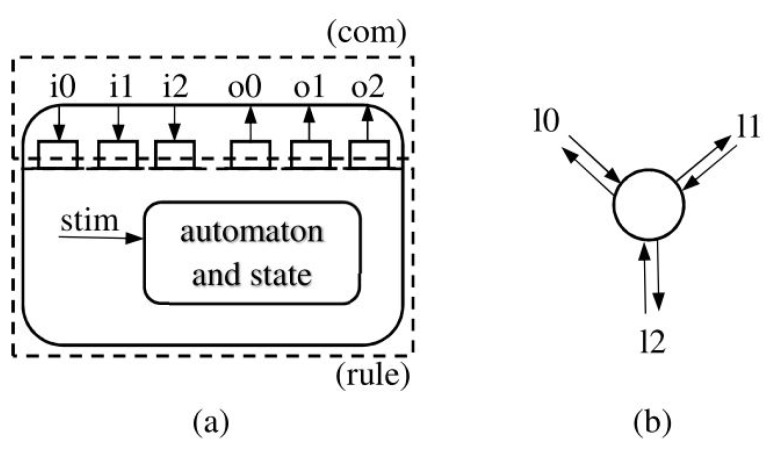
Cell node representation: (**a**) is the internal architecture with an automaton (*rule*) operating on incoming values and local stimuli (*stim*), based on a local set of variables, filling output communication buffers. Communications (*com*) are operated to and from the input and output buffers; (**b**) is the external point of view that only shows bidirectional links of a cell node.

- The synchronous distributed messaging model [43] can support parallel computation by associating cells with communicating processes. In this case, process progress by locked steps, based on messages being sent and received to or from neighbor nodes. The steps are split into two phases, one for communications with the neighborhood, the other to execute the transition rule. Figure 12 shows an internal node representation and the outside connection with three input and output links. This model does not need to specify the relative speed of processes, and can therefore be used for multi-cores or supercomputers.
- Another way to take advantage of parallelism is to use data parallel Single Instruction Multiple Data (SIMD) processors to execute a group of processes simultaneously. Current graphics accelerators propose solutions up to two thousand processors working concurrently, and exchanging data synchronously in shared memory. State spaces must be copied to the accelerator memory. Then, a loop of steps can be run completely on the acceleration, which is very efficient.

Production of code from cell systems to this schema was done for two targets:

1. Asynchronized Occam communicating processes [44,45] targeting KRoC compiler and multi-cores [46],
2. CUDA code production [47] targeting *NVIDIA* tools and accelerators.

### 2.5. Cellular Systems Workflow Organization

Most of the work described in this article is supported by a set of dedicated tools from the University of Bretagne Occidentale (UBO). The design flow can be summarized as follows:

1. **Zone selection** is done by moving a graphical window anywhere, with any level of zoom. The tool extracts graphic tiles, and displays the contents.
2. **Cell segmentation** is obtained by splitting the view into rectangles of a given size specified as a width × height value. Cells will carry an image and a geographical location from the underlying image.
3. **Binding cell together** and producing a cell system implies the choice of a connectivity (Moore, Von Neumann), and possibly filtering cells by colors or elevation. This step also injects external values from a variety of sources, including elevation.
4. **Adding behavior** programs the cellular system at the local level, given a cell system architecture that step 3 (Figure 11) automatically produces.

Steps 1 to 3 are interactive and can be achieved *in minutes*. Step 4 is a concurrent programming activity specific to a concurrent platform which requires elaboration of CA transition rule and coding for the target platform.

Physical phenomena simulation can reveal places of interest for sensing. In the case of flooding, simulation track accumulation and circulation of water.

## 3. Heavy Rain Simulation on a Complex Terrain

To illustrate cell system interactions and behavior, we use the example of a zone *receiving* and *flooding* rain, as discussed in Section 1.4.

### 3.1. Managing Space

Space is defined by geographical coordinate bounds, and a possible selection operated on cells. Selected cells have common properties, such as an elevation above or under a threshold level (Figure 11, step C), some color characteristics, or some signature produced from observed parameters. Thus, the cell system can be rectangular or have arbitrary shapes and isolated subsystems.

Practically, cell systems first appear as arrays of objects that carry geometric coordinates, geographic coordinates, and a pixel array extracted from the original image.

Besides the zone structure, designers need to specify neighborhood, representing local physical influences and producing the necessary connectivity between processes. We can just admit that the space animation will be obtained by messages exchanged between neighbors. Execution software and hardware will allow adaptation to the intermediate communicating process model.

The case of rain reveals several interactions:

1. Millimeters of water falling on the ground. For this simulation, we admit that the quantity can vary over time, but will remain uniform.
2. Water disappearing locally for reasons such as absorption or evaporation.
3. Water passed *locally* from cell to cell according to elevation differences.

**Figure 13:**
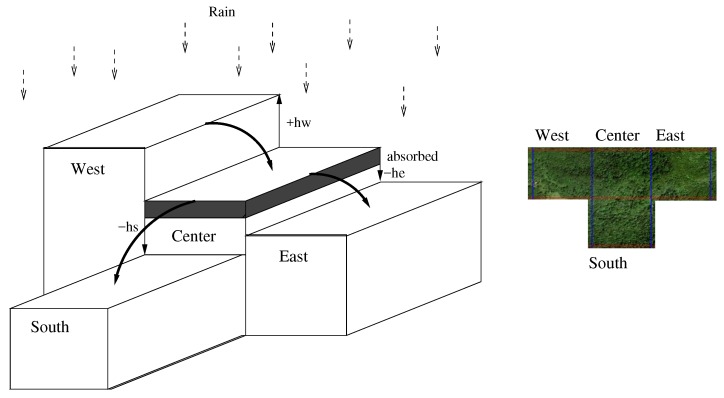
Physical exchange during a rain episode. An incomplete neighborhood from a system shows a center cell with 2 neighbors west and east, and 1 in the south. The physical behavior is water flowing downward, represented here by synchronous messages sending water quantity west to center, and center to the east. Refer to [49] for more realistic behaviors.

Other cases include different specific problems for signal propagation (Section 5), sound propagation [48], or insect swarms behavior [40]. For flood modeling, Von Neumann neighborhood is convenient. Figure 13 shows such a neighborhood, with the North cell lost, and some elevation differences appearing above and below a center cell.

### 3.2. Transition Rule

Each cell will receive rainwater, dispatch part of this water to neighbor cells with lower elevation, and absorb another part. A complete study will take into account the ground specific characteristics, and current weather (ECOCLIMAP [50] supporting French meteorology AROME model provides more than 20 parameters for cells of 1 km × 1 km). While real dependencies are water leaking from cell to cell, the abstract behavior is represented by messages sent and received to and from neighbors. Vertical water behavior is directly encoded in the transition rule that can be specified as follows:

*t*: time *t* represented by a step number,
$Q_{t}$: water quantity in a center cell at the beginning of time step *t*,
$\alpha_{t}$: rainfall at time *t*,
β: percentage of water remaining on each cell after each step,
$cell_{i}.elevation$: elevation value of $cell_{i}$,
$cell_{c}.elevation$: elevation value of center cell,
$\delta_{i}$: the difference of elevation between neighbor $cell_{i}$ and the center cell,
Δ: sum of all elevation differences,
$outF_{i}$: amount of water out coming from center cell to neighbor $cell_{i}$,
$inF_{i}$: amount of water in coming to center cell from neighbor $cell_{i}$.

The water quantity of center cell at time t+1 after local absorption is

(1) $$remaining=Q_{t}\times \beta ,$$

but the center cell receives rainfall within its area the following:

(2) $$rain=\alpha_{t}.$$

The quantity of water coming from neighbors is taken into account

(3) $$received=\sum_{i=1}^{n}inF_{i},$$

where *n* is a number of neighbors of the center cell.

A center cell also has to distribute water to surrounding neighbors in the proportion of elevation differences. This is calculated as follows:

(4) $$\delta_{i}=cell_{c}.elevation-cell_{i}.elevation.$$

It is obvious that the water of a center cell only flows to neighbor cells with lower elevations so that only positive values of $delta_{i}$ are used to compute the total proportion of all differences

(5) $$elevation\;Above=\Delta =\sum \delta_{i},\forall \delta_{i}>0.$$

Equation (6) figures out the amount of water that a cell distributes to its lower neighbors. For higher elevation neighbors, with $\delta_{i}<0$, there is no water flowing out, $outF_{i}=0$:

(6) $$sent_{i}=outF_{i}=Q_{t}\times \left(\delta_{i}/\Delta \right),\forall \delta_{i}>0.$$

Eventually, the center cell updates its own quantity of water by adding and subtracting. The whole system executes a synchronous transition from *t* to t+1:

(7) $$Q_{t+1}:=remaining+rain+received-\sum_{i=1}^{n}sent_{i}.$$

Notice that this transition rule needs to know the neighbor relative elevations $cell_{i}.elevation$. In reality, physical rules will discover these relations naturally. In the case of simulations, a preliminary procedure called *neighborhood discovery* is executed to establish initial knowledge such as the number and the elevations of cells around.

## 4. Parallel Algorithms for Cellular Long-Range Coverage Computations

### 4.1. General Idea

**Figure 14:**
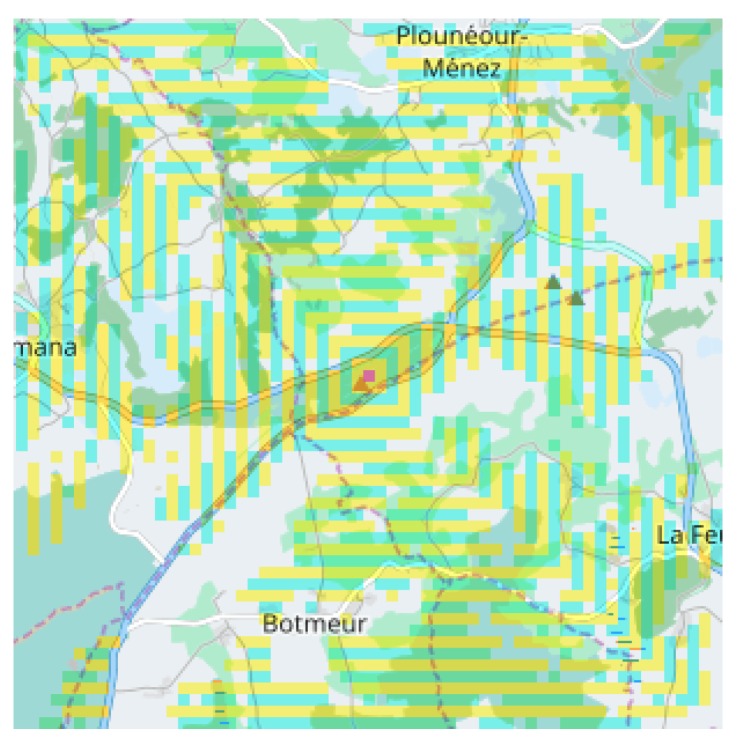
Radio signals propagate in concentric squares step by step. Reachable cells are represented in colored stripes.

Once a geographic space has been selected, a concern is to compute reachable cells in the line-of-sight from an emitting position. According to Section 2.4, we admit that each cell is represented by a process, and simulation is achieved by cycling on a synchronous parallel program: communication with neighbors, updating the local state, and preparing next cycle communications. The *line-of-sight* (LoS) is a ray broadcast in any direction from a root emitter. Propagation will be stopped or modified by ground obstacles such as hills, valley, etc. The simulation parallel algorithm mimics the physical behavior, by propagating the signal inside a spanning tree rooted at the emitter cell, and covering progressively all the space in concentric “*circles*” as shown in Figure 14.

**Figure 15:**
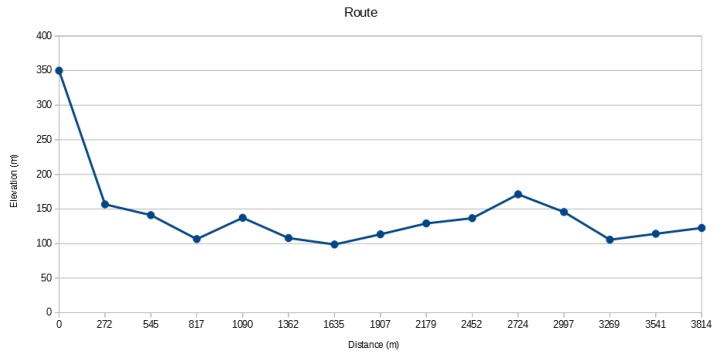
A profile obtained along a route. Let’s assume that an emitter located on the furthest left of the chart, with a distance of 0 m, an elevation of 350 m. According to LoS condition, three points at distances 2997 m, 3269 m and 3541 m seem unable to receive the signal from the emitter.

Each new step in the algorithm adds a new circle, and the computation finishes in 2×log(n) steps where *n* is the number of cells. During ray propagation, the ground profile is collected into a route. A route profile is shown in Figure 15. Routes are completed progressively based on positions and elevations. Thus, each cell can decide if the emitter is visible or not by comparing its slope to root to previous ones in the received profile, as shown in Algorithm 1.

**Algorithm 1** Setup visible nodes from emitter based on the LoS condition.

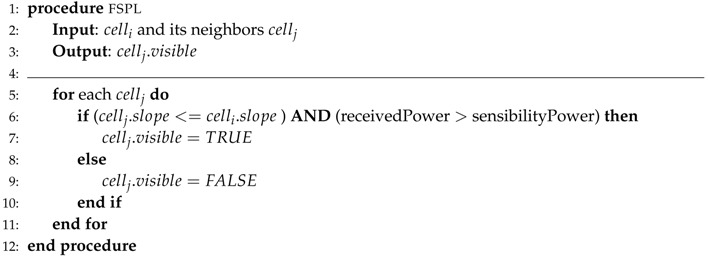



### 4.2. Vertical Model

Signal propagation progresses in steps according to time. It builds progressively a route from the emitter to any other cell, as an array holding traversed locations and signal parameters (Figure 15). As it is the case for most of the cellular simulation algorithms, nodes start with a discovery stage, where geometric coordinates are exchanged to bind communication channels to cardinal directions. This is mandatory to deal with the irregularity of cell system shapes as shown in Figure 11, step (3), and also to maintain horizontal signal direction, as discussed in Section 4.3.

To start the building a propagation, an emitter has to initialize a tracing route with its own identity and geographic location. Other cells start their processes with an empty tracing route. Note that the tracing route, in this system, is the payload of the message for communications within the cell system. The root cell then sends its message to all neighbors via eight directions from the Moore neighborhood. Each cell accepts only one message and then assigns the corresponding owner of the accepted message as its parent. This cell must send an acknowledgment message back to the parent cell to confirm the dependency relationship between them. The next step in the transition rule is to insert its own location into the tracing route, then to pack routes and spread out to neighbors. If *side* is the maximum between *width* and *height*, then, at most, *side* rounds are necessary to cover an entire zone.

### 4.3. Horizontal Model and Directed Breadth-First Search

**Figure 16:**
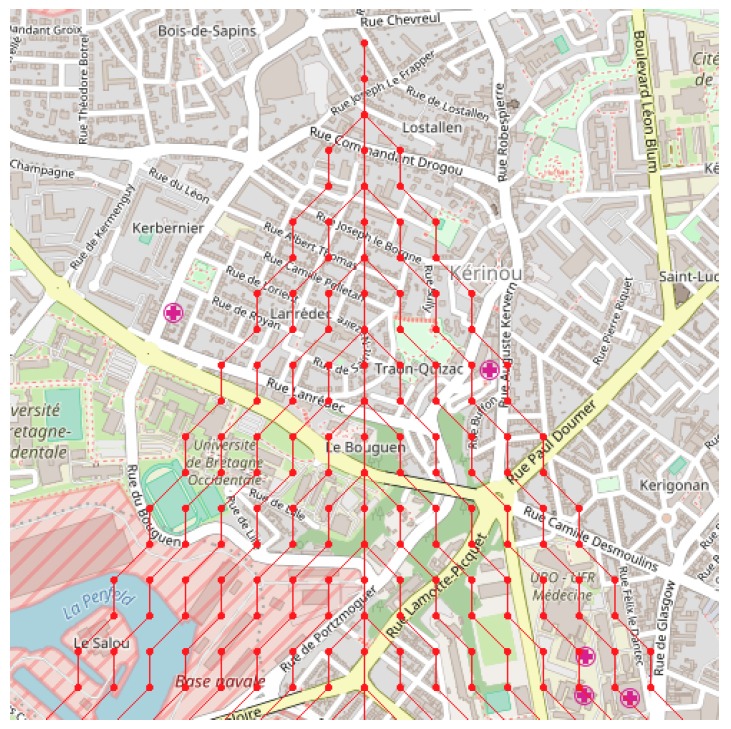
Directed BSF is a distributed parallel algorithm able to manipulate data on grid cells. Its aim is to mimic point-to-point radio links adopting the LoS condition. Segmented lines are laid on a map to represent how cells forward incoming signal gradually from a root cell (*x* = 10, *y* = 1) to definite directions.

The original Breadth-First search (BFS) algorithm builds a tree covering a whole network starting from a root position. For radio propagation, this tree would allow reaching any cell in a minimum number of steps, propagating a route to this cell without managing signal horizontal directions. In the case of radio signals, there is the necessity to guide the route horizontally to maintain the best approximation of a straight line. A sequential algorithm that reduces horizontal errors to a minimum was suggested by Bresenham to draw graphic lines on bitmaps [51]. In our case, it was preferred to find a parallel distributed approach based on Directed BFS (DBFS) that could also match particular geographic or electromagnetic dynamic considerations. Figure 16 shows DBFS routes resulting from this algorithm according to an emitting node and some target receivers.

At the difference of a BFS, the cell transition function makes a decision on receipt of route messages based on the source position and its own source position. It adds its data to the route, and it forwards the route to neighbors of interest. The neighborhood was discovered at the initialization stage, associating link indexes and cardinal directions.

## 5. Radio Signal Propagation on Complex Terrains

*Radio propagation models* are used to describe the qualifications and reliability of links using radio frequency. These models were based on the physics of the diffusion of electromagnetic waves with respect to both constructive and destructive interference of environment. They can be practically applied to parameters inside routes transmitted from cell to cell, according to Section 4.

Free space path loss (FSPL) is the simplest model based on the Friis transmission equation. By assuming unobstructed along the transmission path, FSPL represents the line-of-sight decay of an electromagnetic wave as a function for distance only. This model exposes significant limits for complex terrain areas. Empirical path loss models were proposed to cope with the effects of turbulent terrains. An advantage is that these models attempt to estimate power loss as a function of distance and radio frequency taking into account the terrain heterogeneity as well as the effects of real transmission environment [52,53].

### 5.1. Free Space Path Loss Model

In terms of radio communication, a *Free Space Condition* is a region where there is no obstacle along the propagation path of radio waves. A radio signal is emitted by a transmitter and then it propagates in any direction at the speed of light. In this case, the signal energy can be received by an antenna in inverse proportion of the distance away from the signal source. In addition, the received power depends on the transmitted power, and gains of both transmit and receive antennas. Fading margin and other loss such as system loss, cables, and connectors are also considered. The power of the signal lost on the path is called the *Free Space Loss* represented by

(8) $$FSPL=20log_{10}\left(\frac{4\Pi d}{\lambda }\right).$$

Algorithm 2 is derived from Equation (8) in order to describe how to calculate the attenuation of signal power due to free space path loss. As a consequence, the free space power received is given by Equation (9), the Friis free space equation [54]:

(9) $$P_{r}\left(d\right)=\frac{P_{t}G_{t}G_{r}{\left(\lambda \right)}^{2}}{{\left(4\pi \right)}^{2}d^{2}L},$$

where

$P_{t}$: transmitted power,
$P_{r}\left(d\right)$: received power,
$G_{t}$: transmitter antenna gain,
$G_{r}$: receiver antenna gain,
*d*: distance between transmitter and receiver in meters,
*L*: system loss factor,
λ: wavelength in meters.

**Algorithm 2** Received signal power in the Free Space Path Loss Model.  1: **procedure**
SignalPower  2:   **Input**: txPower, waveLength, distance  3:   **Output**: receivedPower  4:   **for** each neighbor of $cell_{i}$
**do**  5:      **if** neighbor.visible **then**  6:      receivedPower ← txPower × (SQR(waveLen /(4.0 ×valuePi ))× SQR(1/distance))  7:      **end if**  8:   **end for**  9: **end procedure**

### 5.2. Single Knife-Edge Diffraction Model

Diffraction appears around objects such as buildings, vegetation, etc. The losses caused by these obstacles can be described by a *Knife Edge Diffraction* model. This is considered as a function of the path difference around the obstacles and could be explained by Fresnel zones. Diffraction loss of signal after a knife edge is calculated as a function of the Fresnel parameter *v*:

(10) $$v=h(\sqrt{\frac{2}{\lambda }\left(\frac{1}{t_{t}}+\frac{1}{d_{r}}\right)}),$$

where *h* is the height of obstacle. $d_{t}$, $d_{r}$ are the distances from the obstacle to the emitter and the receiver, respectively. Algorithm 3 demonstrates the computation of received power within a shadowing area because of a single knife edge obstacle:

(11) $$L\left(v\right)=6.9+20log\left(\sqrt{{\left(v-0.1\right)}^{2}+1}+v-0.1\right).$$

**Algorithm 3** Received signal power in the Single Knife-Edge Diffraction Model.   1: **procedure**
diffractionLoss   2:   **Input**: fsplPower, h, $d_{1}$, $d_{2}$   3:   **Output**: diffPower   4:   v = h * SQRT((valuePi/2)×((1/$d_{1}$) + (1/$d_{2}$)))   5:   **if** v < 0 **then**   6:      diffLoss ← 0   7:   **else**   8:      **if** v < 2.4 **then**   9:      diffLoss ← 6 + (9 × v) + (1.27 × SQR(v))  10:      **else**  11:      diffLoss ← 13 + 20×$log_{10}\left(v\right)$  12:      **end if**  13:   **end if**  14:   diffPower = fsplPower - diffLoss  15: **end procedure**

In Algorithm 3, *receivedPower* is defined as the signal strength in dBm obtained at a receiver. In addition, *sensibilityPower* is the minimum value of signal strength in which condition a receiver can detect and demodulate a message successfully.

### 5.3. Okumura–Hata Model

This is a dedicated radio propagation model to predict the path loss regarding geographic environments such as urban, suburban, and open areas [53,54]. The formula for the Okumura–Hata model is given by

(12) $$L=69.55+26.16log_{10}\left(f\right)-13.82log_{10}\left(h_{b}\right)-a\left(h_{m}\right)+\left(44.9-6.55log_{10}\left(h_{b}\right)\right)log_{10}\left(d\right)-K,$$

where

$f_{MHz}$: center carrier frequency of transmission band in MHz.
$h_{b}$: antenna height of base station in meter,
$h_{m}$: antenna height of mobile node in meter,
$d_{km}$: distance in kilometers,
$a(h_{m})$ for each type of area and *K*, see Table 2.

The model is valid for radios using carrier frequencies from 150 to 1500 MHz with an effective height of antennas from 30 m to 1000 m and distances ranging from 1 km to 100 km. Table 2 presents list of $a(h_{m})$ and *K* values for different terrain types.

### 5.4. Correctness of the Different Radio Communication Models

**Figure A5:**
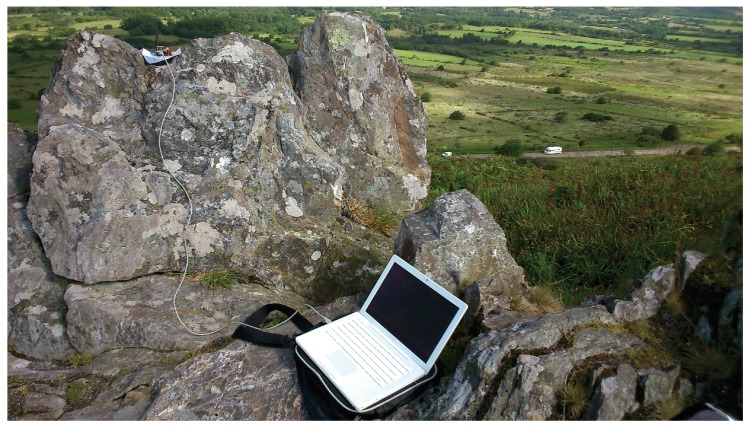
A base station is equipped with a LoRa board (emitter) and a MacBook. This base station was deployed on the top of the Roc’h Trevezel mountain, France. The receiver is another LoRa board mounted on a car moving around.

The terrain was a mountain area investigated by applying the three models during simulations. The computation results are shown in Table 3. Figure A5 displays the coverage obtained by the model of *single knife-edge diffraction*, and effective coverage measured by a mobile.

## 6. Conclusions

To optimize communication ranges, two major concerns are radio waves blocked by obstacles and propagation loss in proportion with a point-to-point distance. Terrains to be covered can be classified and partitioned as smooth, or rough according to metrics (Section 1.2). In smooth terrain areas, radio transmission can be considered as a near line-of-sight (NLoS). As a result, the issue of topologies can be almost neglected and the communication distance is expressed in relation to power reduction along the path. The free space path loss (FSPL) equation was proposed for this aim (Section 5). For example, using LoRa technology for a point-to-point connection in NLoS conditions, the distance for transceiving data is expected to reach hundreds of kilometers [55].

In complex terrain areas, heterogeneity of topologies seriously impacts the quality of radio links, especially for low power and long-range communication networks. Different propagation models allow for representing the median of the expected path loss such as the Longley–Rice model, the ITU model, and the Okumura–Hata model, as described in Section 5.

Appendices give details on experimental measurements, with the communication distance checked to be around 5 km in complex urban area and up to 20 km in rural and shore ones. Experiment results shown in Table A1 confirm the interest of a computer-aided approach. It may be concluded that communication in a LoRa network is strongly dependent on the considered environment. Hence, the actual coverage prediction must take topographic complexity into account as a critical factor. An assessment of physical simulation was also conducted for flash flooding problems (Section 3). This algorithm was applied to the practical case of an intense rain found on 3 June 2018, and compared with flooding observations. The rain occurred in a rough terrain urban area and the simulation was able to retrieve major flooding places and level of water. There is a major interest in these kinds of physical simulations that reveal places of interest for sensing and establish causality between events.

The software developed in this project is easy to use for cellular system generation. Implementing cellular transition rules, tuning and verifying these rules necessitate in-depth investigations. Several domains were investigated, from sound propagation to insect behaviors. Thus, the methodology appears very general and flexible (see Table 4). Parallel programs implement described algorithms very efficiently (Table 5 and Table 6). Efficiency is mandatory if space exploration strategies are to be developed.

Several research opportunities are created by this work:

Synthesizing long-range mesh radio networks would allow for covering very large surfaces and require optimization of relay placement. Powerful parallel algorithms can help to overcome the coverage problem complexity.
Line-of-sight propagation and water spreading are examples of common spatial behaviors found in nature. Many biological, mechanical or physical processes have similar properties. Sensing of environment could take benefits from generic frameworks helping to develop and compose such simulations.
Applicability for large problems available for the public can be obtained by publishing web interfaces to services dependent on two separated concerns: zone selection and cell resolution, and cellular system libraries.

## Figures and Tables

**Table 1:** Geo-locations of points where values of water level were recorded.

Point	Color	Latitude	Longitude	Elevation (m)
P1	Red	36.589895361	4.925651550	554.7
P2	Green	36.587414366	4.925651550	536.8

**Table 2:** List of $a(h_{m})$ and *K* values for each type of area [54].

Type of Area	a$\left(h_{m}\right)$	K
Open		$4.78{\left[log_{10}\left(f\right)\right]}^{2}-18.33log_{10}\left(f\right)+40.94$
Suburban	$\left[1.1log_{10}\left(f\right)-0.7\right]h_{m}-\left[1.56log_{10}\left(f\right)-0.8\right]$	$2{\left[log_{10}\left(f/28\right)\right]}^{2}+5.4$
Small city		0
Large city	$3.2{\left[log_{10}\left(11.75h_{m}\right)\right]}^{2}+4.97$	0

**Table 3:** Comparison in number of points found in coverage from a total of 96,431 points.

Model	Points
Free space path loss	19,537
Single knife-edge diffraction	22,305
Okumura-Hata	17,621

**Table 4:** A characterization of space and signal relations.

Datagrid	Model	Resolution (m)
SRTM30	2D/3D	30 (https://lta.cr.usgs.gov/SRTM1Arc)
SRTM90	2D/3D	90 (https://blogs.esri.com/esri/arcgis/2015/06/26/terrain-3d-now-with-global-srtm-30-meter-content/)
Weather broadcast	2D/3D	3000 (https://www.ncdc.noaa.gov/data-access/model-data/model-datasets/numerical-weather-prediction)
LiDAR	3D	5 (https://content.meteoblue.com/en/research-development/data-sources/nmm-modelling/model-domain)
Coastal ocean	2D/3D	1200 (https://pdfs.semanticscholar.org/55be/a487827c28aaaf713017c499e4f33aed62fd.pdf)
Earth magnetic	2D/3D	3700 (http://www.sciencedirect.com/science/article/pii/S0377042798002465)
Ocean acoustics	2D/3D	2500 (https://www.ngdc.noaa.gov/geomag/emag2.html)
		0.5 (Peter Wille. Chapter 5: The Sea Floor—Natural Formations.
Road traffic noise	2D/3D	In *Sound Images of the Ocean: in Research and Monitoring*.
		Springer-Verlag Berlin Heidelberg, 2005. ISBN 978-3-540-27910-5)
Pickcell	2D/2.5D	30

**Table 5:** Execution times for three processes. There are 58,725 cells in a regular squared grid with a cell size 3 × 3 pixels corresponding to actual area 115 × 115 m.

Processes	Number of Rounds	Time (s)
Compute ruggedness index	1	0.026
Flood simulation	32	0.103
Coverage prediction	261	0.211

**Table 6:** Execution times for flood simulations which are performed on Linux Ubuntu PCs with Intel® Core™ i3-4005U CPU @ 1.70GHz × 4, 8 GiB DDRAM, card NVIDIA GeForce 820M (96 CUDA Cores), Intel® Core™ i7 CPU 920 @ 2.67GHz × 8, 4 GiB DDRAM, card NVIDIA GeForce GTX 680 (1536 CUDA Cores) and Intel® Core™ i7-7700K CPU @ 4.20GHz × 8, 16 GiB DDRAM, card NVIDIA GeForce GTX 1070 (1920 CUDA Cores).

			Execution Time	
Resolution (Pixels)	Number of Cells	820M	GTX 680	PGTX 1070
3 × 3	58,725	28.168 (ms)	6.5269 (ms)	1.3373 (ms)
5 × 5	21,060	10.411 (ms)	2.1728 (ms)	518.64 (μs)
10 × 10	5226	2.5234 (ms)	454.59 (μs)	83.696 (μs)
15 × 15	2340	1.1155 (ms)	181.48 (μs)	65.916 (μs)
20 × 20	1287	602.47 (μs)	171.19 (μs)	62.085 (μs)

**Figure A1:**
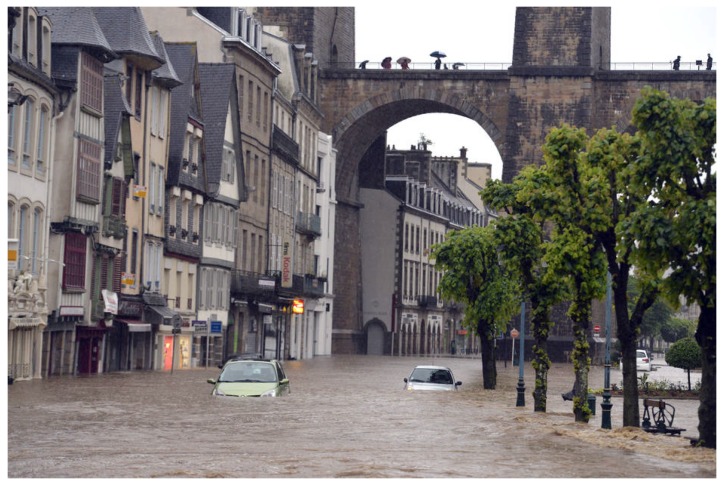
Heavy flooding in the center of Morlaix, France due to a storm on 3 June 2018 (Photo: Le Télégramme).

**Figure A2:**
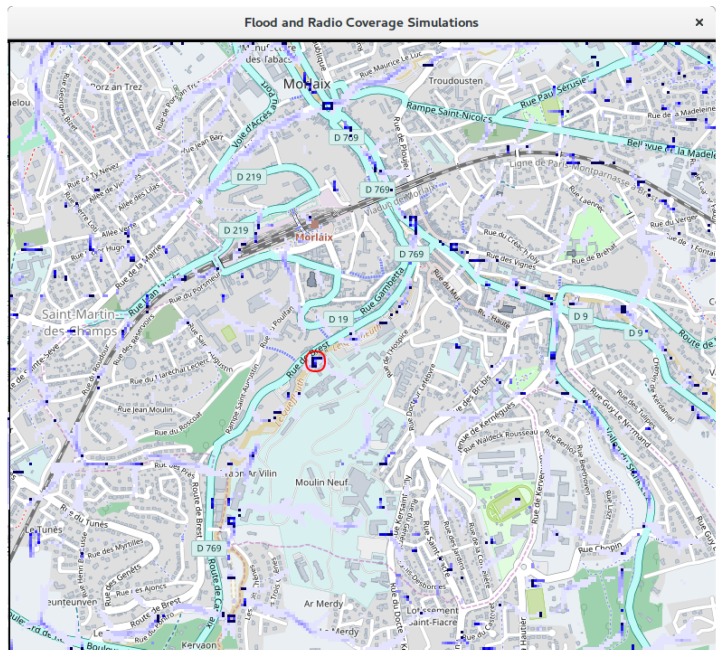
Simulation results for Morlaix, obtained by executing massively parallel processes on GPUs with thousands of CUDA cores. The experimental zone is comprised of 58,275 cells corresponding to actual area 3 × 3 km. A place of concern is indicated by a red circle. This is *rue de Brest* near the river *Queffleuth* (longitude: −3.8329839706421, latitude: 48.573767695437, elevation: 10.1 m) where the water level went up to 60 cm according to *Le Télégramme* newspaper. The main flooding directions can be seen in light blue over the maps.

**Figure A3:**
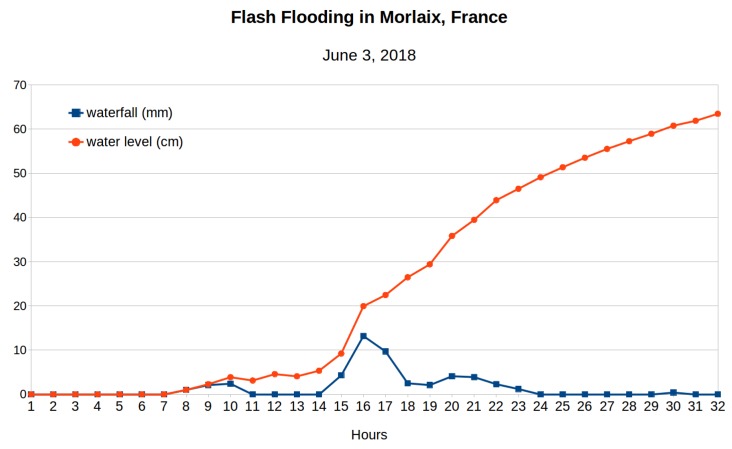
Chart shows rainfall levels over 32 h from 12:00 a.m. on 3 June 2018, to 8:00 a.m. on 4 June 2018. It was noticed that serious flooding often comes several hours after a storm or heavy rain. After the rain stopped, it required a couple of hours or days to drain all of the water.

**Figure A4:**
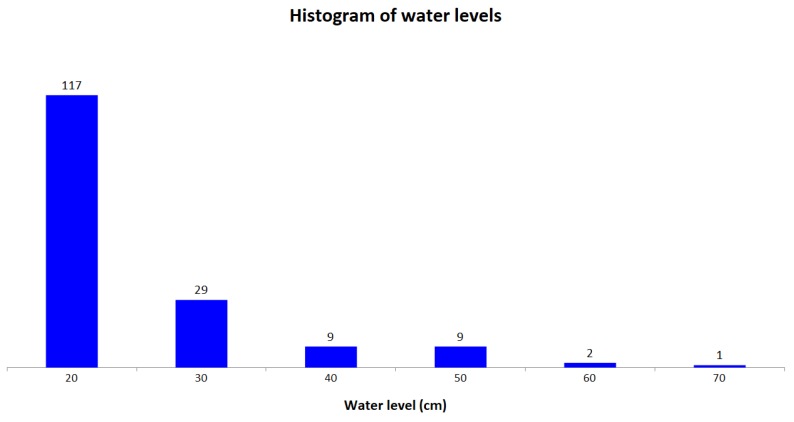
Even though the heavy rain just occurred during a few hours (3:00–5:00 p.m.), simulation results show that there are 50 points where water levels are from 30 to 70 cm.

**Figure A6:**
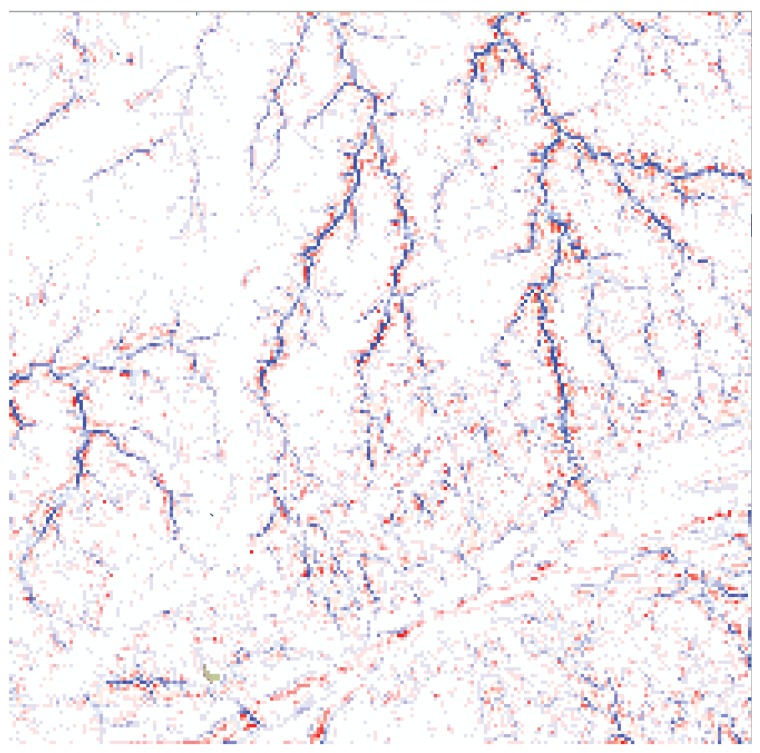
Terrain complexity analysis for the Roc’h Trevezel using TPI metrics. This shows remarkable landform splits in the North–South direction. This provides critical references for network deployment aiming at enhancing the system robustness and range and reducing the cost as well.

**Figure A7:**
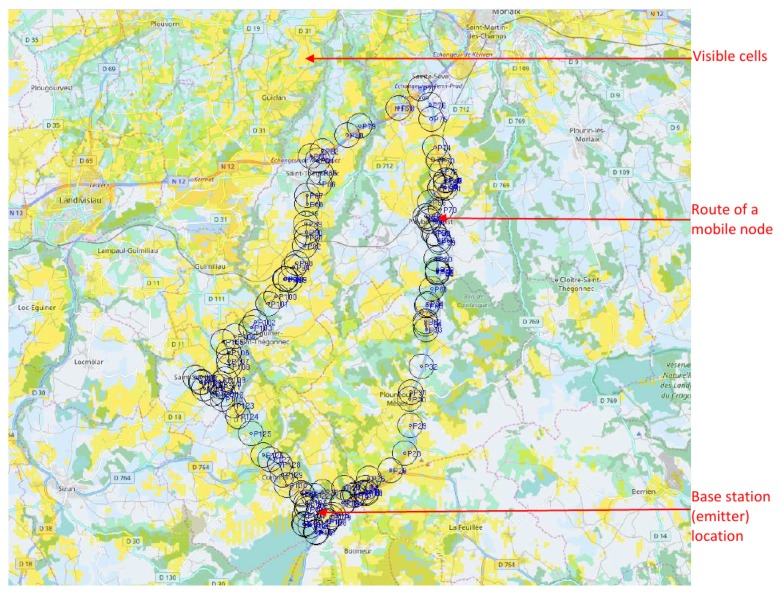
The base station (emitter) is located on top of the mountain (latitude: 48.4051306, longitude: −3.9077762, elevation: 345 m). A receiver is placed on a car (receiver) traveling around the area with many hills, valleys, and big trees. From simulation results, places (cells) are able to receive the radio signal highlighted in yellow. Blue circles show points where the receiver successfully obtained messages in our actual measurement.

**Figure A8:**
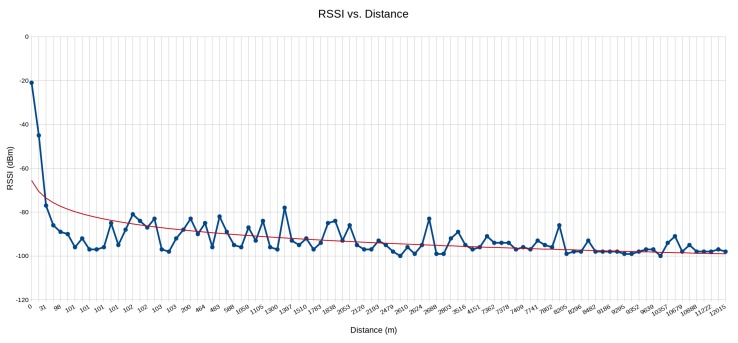
Obtained received signal strength indicator (RSSI) values for experiment in our actual measurement at the Roc’h Trevezel. RSSI values decay as a negative exponential function of distance. The complex terrains in this area seriously impact the quality of radio links.

**Figure A9:**
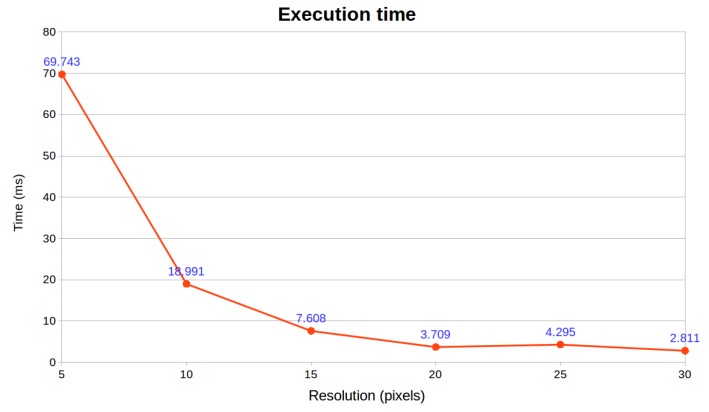
Chart of radio coverage execution times for different resolutions of cell from 5 to 30 pixels. This statistics was recorded for the performance on a PC equipped Core i7-7700K CPU@4.20GHz × 8, 16 GiB DDRAM, card NVidia GeForce GTX 1070 (1920 CUDA cores).

## Appendix A

### Appendix A.1. A Study of a Flash Flood Event

A case study for flash flooding prediction due to heavy rain during a storm on Monday, 3 June 2018, in the town of Morlaix, in France [57]. The objective is for validating the accuracy of our proposed tool for flash flooding, especially for complex terrain areas.

Apart from a data grid produced by QuickMap/NetGen/PickCell, to execute flooding simulation, it is necessary for the rainfall values to be measured each hour during a storm, hurricane, etc. as input data (see the blue line in Figure A3. The data can be retrieved from the database of weather broadcast services such as meteofrance.com, meteoblue.com, etc.

Our proposed simulator can be efficient to predict risk areas where flooding due to heavy rain may occur.

In fact, flood simulation utilized the frame tool flow as what was being used for radio signal propagation simulation and radio coverage prediction as explained in Section 3. For this study, the time step was one hour, and we keep β at 20%, $\alpha_{t}$ rainfalls were taken from public websites shortly after the episode.

It leverages further intensive studies for reducing the risk of natural disasters, especially in complex topographical areas. It gives many benefits to the local residents’ lives in terms of human aspects, as well as economic activities, by informing about the risks of their position regarding exceptional events.

### Appendix A.2. Experimental Measurements of Radio Coverage

Some case studies were investigated with the credible installation position for *base stations* (BS) controlling hundreds of sensors. In each case, the signal strength was recorded with a picture showing the BS environment. To evaluate the impact of transmission environment on reliability and robustness of radio links in the context of wide-range wireless networks, several places with different terrains in Brittany, in France, were selected for our intensive investigations. The case as shown in Figure A5 required a visit to the *Arrée Mountains* in central Brittany. These mounts culminate at less than 400 m and are the sites chosen for TV/Radio antennas. The car was driven in a loop for up to 10 km, showing a good accuracy of simulation prediction.

**Table A1:** Three areas with different complexity terrains. *Resolution* and *Actual size* columns give the size in pixels associated with actual size in meters for each cell in regular grid data. The maximum communication in each experiment is shown by the column *Range*. The *Match* and *Mismatch* column provides a number of visible points which are right matched and mismatched, respectively, between simulation results and obtained values in real measurements.

Area Name	Description	Resolution	Actual Size	Range	Match	Mismatch	% Error
Albert 1^*er*^	Urban area	5×5 pixels	24 m	3 km	64	13	20.21%
Plougastel	River and its banks	5×5 pixels	96 m	9 km	129	4	3.10%
Trevezel	mountain area	5×5 pixels	191 m	11 km	45	8	17.78%
